# 3-(4-Fluoro­phen­yl)-6-meth­oxy-2-(4-pyrid­yl)quinoxaline

**DOI:** 10.1107/S1600536809022119

**Published:** 2009-06-20

**Authors:** Hartmut Jahns, Pierre Koch, Dieter Schollmeyer, Stefan Laufer

**Affiliations:** a Institute of Pharmacy, Department of Pharmaceutical and Medicinal Chemistry, Eberhard-Karls-University Tübingen, Auf der Morgenstelle 8, 72076 Tübingen, Germany; b Department of Organic Chemistry, Johannes Gutenberg-University Mainz, Duesbergweg 10-14, D-55099 Mainz, Germany

## Abstract

In the title compound, C_20_H_14_FN_3_O, the quinoxaline system makes dihedral angles of 32.38 (7) and 48.04 (7)° with the 4-fluoro­phenyl and pyridine rings, respectively. The 4-fluoro­phenyl ring makes a dihedral angle of 57.77 (9)° with the pyridine ring. In the crystal, the mol­ecules form dimeric C—H⋯N hydrogen-bonded *R*_2_^2^(20) ring motifs lying about crystallographic inversion centers. The dimeric units stack *via* π–π inter­actions between methoxy­phenyl rings and pyridine–fluoro­phenyl rings with centroid–centroid distances of 3.720 (1) and 3.823 (1) Å, respectively. The respective average perpendicular distances are 3.421 and 3.378 Å, with dihedral angles between the rings of 1.31 (9) and 11.64 (9)°.

## Related literature

Many chinoxaline derivatives have been prepared and their biological activity have been studied, see: He *et al.* (2003 ▶); Kim *et al.* (2004 ▶). For inter­molecular C—H⋯N hydrogen bonds, see: Taylor & Kennard (1982 ▶). For distinct ring motifs formed *via* O—H⋯N hydrogen bonds, see: Habib & Janiak (2008 ▶); Friščič & MacGillivray (2003 ▶). For graph-set notation, see: Bernstein *et al.* (1995 ▶).
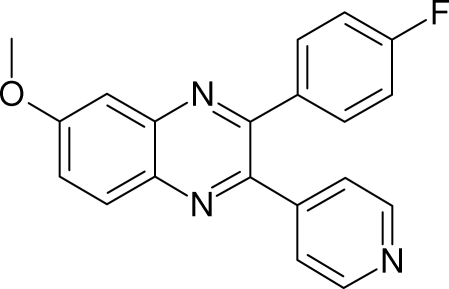


## Experimental

### 

#### Crystal data

C_20_H_14_FN_3_O
*M* *_r_* = 331.34
Orthorhombic, 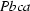

*a* = 7.3886 (4) Å
*b* = 12.2071 (8) Å
*c* = 34.562 (6) Å
*V* = 3117.3 (6) Å^3^
*Z* = 8
Cu *K*α radiation
μ = 0.80 mm^−1^
*T* = 193 K
0.45 × 0.22 × 0.13 mm

#### Data collection

Enraf–Nonius CAD-4 diffractometer
Absorption correction: none
2950 measured reflections
2950 independent reflections
2542 reflections with *I* > 2σ(*I*)
3 standard reflections frequency: 60 min intensity decay: 2%

#### Refinement

*R*[*F* ^2^ > 2σ(*F* ^2^)] = 0.044
*wR*(*F* ^2^) = 0.124
*S* = 1.05
2950 reflections
228 parameters
H-atom parameters constrained
Δρ_max_ = 0.32 e Å^−3^
Δρ_min_ = −0.24 e Å^−3^

### 

Data collection: *CAD-4 Software* (Enraf–Nonius, 1989 ▶); cell refinement: *CAD-4 Software*; data reduction: *CORINC* (Dräger & Gattow, 1971 ▶); program(s) used to solve structure: *SIR97* (Altomare *et al.*, 1999 ▶); program(s) used to refine structure: *SHELXL97* (Sheldrick, 2008 ▶); molecular graphics: *PLATON* (Spek, 2009 ▶); software used to prepare material for publication: *PLATON*.

## Supplementary Material

Crystal structure: contains datablocks I, global. DOI: 10.1107/S1600536809022119/si2176sup1.cif

Structure factors: contains datablocks I. DOI: 10.1107/S1600536809022119/si2176Isup2.hkl

Additional supplementary materials:  crystallographic information; 3D view; checkCIF report

## Figures and Tables

**Table 1:** Hydrogen-bond geometry (Å, °)

*D*—H⋯*A*	*D*—H	H⋯*A*	*D*⋯*A*	*D*—H⋯*A*
C15—H15⋯N21^i^	0.95	2.44	3.368 (3)	165

Symmetry code: (i) 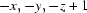.

**Fig. 1.**
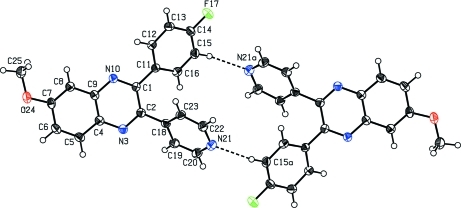
View of the centrosymmetric dimer of I. Displacement ellipsoids are drawn at the 50% probability level. H atoms are depicted as circles of arbitrary size. Hydrogen bonds with dashed lines. Symmetry code a: -x, -y, 1-z.

**Fig. 2.**
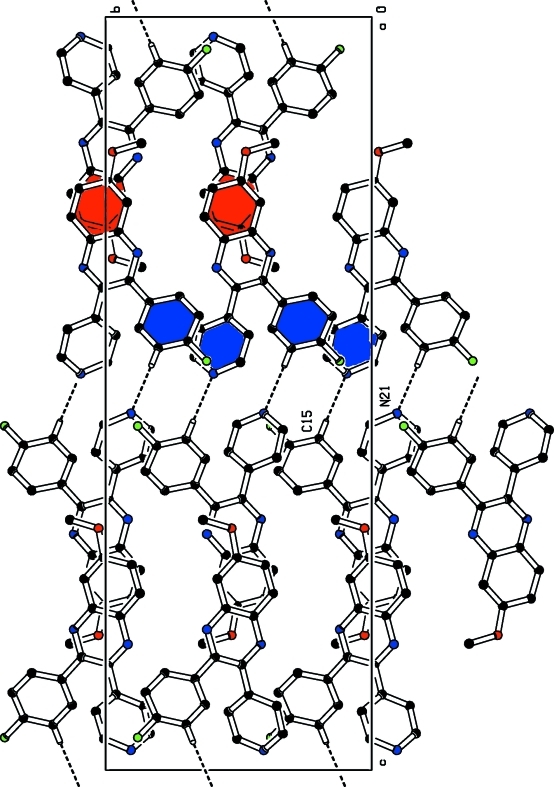
A section of the crystal structure of the title compound, viewed along the b axis. Aromatic rings involved in π—π stacking interactions are shown in red and blue. Hydrogen bonds with dashed lines.

## supplementary crystallographic information

### Comment

Functionalized quinoxaline derivatives are well known in pharmaceutical industry. They have been shown to possess antibacterial activity (Kim *et al*. 2004) and as PDGF-*R* tyrosine kinase inhibitors (He *et al.* 2003).

The title compound, (I), was prepared in the course of our studies on 2-(2-alkylaminopyridin-4-yl)-3-(4-fluorophenyl)quinoxalines as potent p38 mitogen-activated protein (MAP) kinase inhibitors. The two molecules of I are connected into a centrosymmetric dimer by intermolecular C15–H15···N21 hydrogen bonds [graph set *R*_2_^2^(20) (Bernstein *et al.* 1995)] (Fig.1 and Table 1). By searching the CCDC a similar O—H···N hydrogen bond pattern could be found in the structure EHOTUQ (Friščič & MacGillivray 2003), with a *R*_4_^4^(46) ring system. A variety of distinct ring motifs formed *via* hydrogen bonded donors and acceptors (O—H···O, O—H···N) has been described for the 4/1/2 adduct of benzene-1,3,5-tricarboxylic acid, 1,2-bis(1,2,4-triazol-4-yl)ethane and water by Habib & Janiak (2008). The C—H···N hydrogen bond of the title compound (Table 1) confirms the hydrogen-bond geometry values reviewed by Taylor & Kennard (1982), where the C—H···N distances vary between 2.523 Å and 2.721 Å, and the angles around the H atom range between 124.6° and 157.3°. The quinoxaline ring makes dihedral angles of 32.38 (7)° and 48.04 (7)° to the 4-fluorophenyl ring and the pyridine ring, respectively. The 4-fluorophenyl ring makes dihedral angles of 57.77 (9)° with the pyridine ring.

π—π interactions between the pyridin rings and the 4-fluorophenyl rings along the *b* axis have Cg2···Cg4^ii^ distances of 3.823 (1) Å, and the distances between Cg3···Cg3^iii^ of the methoxyphenyl rings are 3.720 (1) Å along the *a* axis (Fig. 2). The respective average perpendicular stacking distances are 3.378 Å and 3.421 Å, with dihedral angles between the rings 1.31° and 11.64°. Symmetry codes ii = 1/2 - *x*, 1/2 + *y*, *z*; iii = -1/2 + *x**y*, 3/2 - *z*. Cg2, Cg3 and Cg4 are the centroids of rings N21, C20, C19, C18, C23, C22; C4 - C9; and C11 - C16.

### Experimental

The title compound I was prepared by irradiating 1-(4-fluorophenyl)-2-(pyridin-4-yl)ethane-1,2-dion (137 mg, 0.6 mmol), *o*-phenylendiamine (82 mg, 0.6 mmol) and methanol-acetic acid (9:1, 6 ml) in a sealed tube at 433 K for 5 min by moderating the initial microwave power (250 W). After the mixture was cooled to room temperature in a stream of compressed air, the solvent was removed under reduced pressure and the residue was purified by flash chromatography (silica gel, from petroleum ether/ ethyl acetate 2:1 to 1:2) to yield 82 mg of I. Crystals suitable for X-ray analysis were obtained by slow crystallization from diethylether/n-hexane.

### Refinement

Hydrogen atoms attached to carbons were placed at calculated positions with C—H = 0.95 Å (aromatic) or 0.98–0.99 Å (*sp*^3^ C-atom). All H atoms were refined in the riding-model approximation with isotropic displacement parameters (set at 1.2–1.5 times of the *U*_eq_ of the parent atom). The structure was solved using a preliminary data collection set. The final measurement on CAD4-diffractometer covered only 1/8 (unique reflection) of the reflection sphere, thus *R*_int_ = 0.0000.

### Crystal data

**Table:**

C_20_H_14_FN_3_O	*F*(000) = 1376
*M**_r_* = 331.34	*D*_x_ = 1.412 Mg m^−^^3^
Orthorhombic, *P**b**c**a*	Cu *K*α radiation, λ = 1.54178 Å
Hall symbol: -P 2ac 2ab	Cell parameters from 25 reflections
*a* = 7.3886 (4) Å	θ = 61–69°
*b* = 12.2071 (8) Å	µ = 0.80 mm^−^^1^
*c* = 34.562 (6) Å	*T* = 193 K
*V* = 3117.3 (6) Å^3^	Plate, colourless
*Z* = 8	0.45 × 0.22 × 0.13 mm

### Data collection

**Table:**

Enraf–Nonius CAD-4 diffractometer	*R*_int_ = 0.0000
Radiation source: FR571 rotating anode	θ_max_ = 70.1°, θ_min_ = 2.6°
graphite	*h* = 0→8
ω/2θ scans	*k* = 0→14
2950 measured reflections	*l* = 0→42
2950 independent reflections	3 standard reflections every 60 min
2542 reflections with *I* > 2σ(*I*)	intensity decay: 2%

### Refinement

**Table:**

Refinement on *F*^2^	Secondary atom site location: difference Fourier map
Least-squares matrix: full	Hydrogen site location: inferred from neighbouring sites
*R*[*F*^2^ > 2σ(*F*^2^)] = 0.044	H-atom parameters constrained
*wR*(*F*^2^) = 0.124	*w* = 1/[σ^2^(*F*_o_^2^) + (0.063*P*)^2^ + 1.8096*P*] where *P* = (*F*_o_^2^ + 2*F*_c_^2^)/3
*S* = 1.05	(Δ/σ)_max_ < 0.001
2950 reflections	Δρ_max_ = 0.32 e Å^−^^3^
228 parameters	Δρ_min_ = −0.24 e Å^−^^3^
0 restraints	Extinction correction: *SHELXL97* (Sheldrick, 2008), Fc^*^=kFc[1+0.001xFc^2^λ^3^/sin(2θ)]^-1/4^
Primary atom site location: structure-invariant direct methods	Extinction coefficient: 0.0024 (2)

### Special details

**Table:**

Geometry. All e.s.d.'s (except the e.s.d. in the dihedral angle between two l.s. planes) are estimated using the full covariance matrix. The cell e.s.d.'s are taken into account individually in the estimation of e.s.d.'s in distances, angles and torsion angles; correlations between e.s.d.'s in cell parameters are only used when they are defined by crystal symmetry. An approximate (isotropic) treatment of cell e.s.d.'s is used for estimating e.s.d.'s involving l.s. planes.
Refinement. Refinement of *F*^2^ against ALL reflections. The weighted *R*-factor *wR* and goodness of fit *S* are based on *F*^2^, conventional *R*-factors *R* are based on *F*, with *F* set to zero for negative *F*^2^. The threshold expression of *F*^2^ > σ(*F*^2^) is used only for calculating *R*-factors(gt) *etc*. and is not relevant to the choice of reflections for refinement. *R*-factors based on *F*^2^ are statistically about twice as large as those based on *F*, and *R*- factors based on ALL data will be even larger.

### Fractional atomic coordinates and isotropic or equivalent isotropic displacement parameters (Å^2^)

**Table:**

	*x*	*y*	*z*	*U*_iso_*/*U*_eq_	
C1	0.0332 (2)	0.08992 (13)	0.65003 (5)	0.0212 (4)	
C2	0.1240 (2)	−0.01069 (13)	0.64015 (5)	0.0234 (4)	
N3	0.1695 (2)	−0.08283 (11)	0.66708 (4)	0.0248 (3)	
C4	0.1415 (2)	−0.05500 (14)	0.70477 (5)	0.0241 (4)	
C5	0.1912 (2)	−0.12816 (15)	0.73464 (5)	0.0280 (4)	
H5	0.2383	−0.1984	0.7284	0.034*	
C6	0.1717 (3)	−0.09798 (15)	0.77228 (5)	0.0290 (4)	
H6	0.2040	−0.1478	0.7922	0.035*	
C7	0.1034 (2)	0.00738 (15)	0.78208 (5)	0.0257 (4)	
C8	0.0529 (2)	0.07962 (14)	0.75376 (5)	0.0253 (4)	
H8	0.0074	0.1499	0.7604	0.030*	
C9	0.0691 (2)	0.04866 (14)	0.71462 (5)	0.0224 (4)	
N10	0.01133 (19)	0.11909 (11)	0.68664 (4)	0.0228 (3)	
C11	−0.0457 (2)	0.16507 (13)	0.62087 (5)	0.0214 (4)	
C12	−0.0501 (2)	0.27756 (14)	0.62867 (5)	0.0240 (4)	
H12	0.0020	0.3047	0.6519	0.029*	
C13	−0.1297 (3)	0.34958 (14)	0.60292 (5)	0.0277 (4)	
H13	−0.1341	0.4258	0.6084	0.033*	
C14	−0.2026 (2)	0.30841 (15)	0.56913 (5)	0.0277 (4)	
C15	−0.2047 (3)	0.19825 (15)	0.56049 (5)	0.0286 (4)	
H15	−0.2590	0.1720	0.5374	0.034*	
C16	−0.1249 (2)	0.12698 (14)	0.58669 (5)	0.0258 (4)	
H16	−0.1240	0.0507	0.5813	0.031*	
F17	−0.27461 (17)	0.37943 (9)	0.54317 (3)	0.0401 (3)	
C18	0.1841 (2)	−0.03833 (14)	0.60030 (5)	0.0239 (4)	
C19	0.1592 (3)	−0.14261 (14)	0.58520 (5)	0.0286 (4)	
H19	0.0981	−0.1973	0.5998	0.034*	
C20	0.2249 (3)	−0.16553 (15)	0.54863 (5)	0.0340 (4)	
H20	0.2038	−0.2367	0.5385	0.041*	
N21	0.3161 (2)	−0.09438 (13)	0.52658 (5)	0.0344 (4)	
C22	0.3408 (3)	0.00561 (15)	0.54154 (5)	0.0317 (4)	
H22	0.4053	0.0580	0.5266	0.038*	
C23	0.2776 (2)	0.03680 (14)	0.57757 (5)	0.0272 (4)	
H23	0.2979	0.1091	0.5867	0.033*	
O24	0.09436 (18)	0.02718 (11)	0.82084 (3)	0.0324 (3)	
C25	0.0370 (3)	0.13400 (17)	0.83218 (6)	0.0356 (5)	
H25A	0.1183	0.1888	0.8209	0.053*	
H25B	0.0400	0.1398	0.8605	0.053*	
H25C	−0.0866	0.1468	0.8230	0.053*	

### Atomic displacement parameters (Å^2^)

**Table:**

	*U*^11^	*U*^22^	*U*^33^	*U*^12^	*U*^13^	*U*^23^
C1	0.0199 (8)	0.0226 (8)	0.0211 (8)	−0.0022 (6)	0.0009 (6)	−0.0003 (6)
C2	0.0229 (8)	0.0223 (8)	0.0248 (8)	−0.0015 (7)	0.0009 (7)	0.0012 (6)
N3	0.0253 (7)	0.0243 (7)	0.0249 (7)	0.0000 (6)	0.0026 (6)	0.0024 (6)
C4	0.0201 (8)	0.0264 (8)	0.0259 (8)	−0.0007 (7)	0.0018 (6)	0.0031 (7)
C5	0.0270 (9)	0.0260 (8)	0.0311 (9)	0.0018 (7)	0.0026 (7)	0.0058 (7)
C6	0.0267 (9)	0.0313 (9)	0.0289 (9)	0.0013 (8)	−0.0004 (7)	0.0100 (7)
C7	0.0207 (8)	0.0355 (10)	0.0209 (8)	−0.0039 (7)	0.0012 (7)	0.0049 (7)
C8	0.0237 (8)	0.0265 (8)	0.0257 (8)	−0.0004 (7)	0.0014 (7)	0.0011 (7)
C9	0.0184 (8)	0.0254 (8)	0.0234 (8)	−0.0022 (7)	0.0004 (6)	0.0038 (7)
N10	0.0225 (7)	0.0237 (7)	0.0221 (7)	−0.0001 (6)	0.0002 (6)	0.0013 (6)
C11	0.0195 (8)	0.0225 (8)	0.0220 (8)	−0.0002 (6)	0.0024 (6)	−0.0002 (6)
C12	0.0225 (8)	0.0248 (8)	0.0246 (8)	−0.0003 (6)	0.0000 (7)	−0.0019 (7)
C13	0.0293 (9)	0.0226 (8)	0.0312 (9)	0.0019 (7)	0.0014 (7)	−0.0001 (7)
C14	0.0258 (9)	0.0310 (9)	0.0263 (9)	0.0045 (7)	−0.0004 (7)	0.0068 (7)
C15	0.0283 (10)	0.0354 (9)	0.0221 (8)	0.0011 (8)	−0.0029 (7)	−0.0020 (7)
C16	0.0258 (9)	0.0245 (8)	0.0272 (8)	−0.0004 (7)	0.0006 (7)	−0.0031 (7)
F17	0.0459 (7)	0.0397 (6)	0.0347 (6)	0.0119 (5)	−0.0078 (5)	0.0092 (5)
C18	0.0219 (8)	0.0235 (8)	0.0262 (9)	0.0030 (7)	−0.0012 (7)	0.0001 (7)
C19	0.0318 (10)	0.0241 (9)	0.0300 (9)	−0.0010 (7)	0.0004 (8)	0.0014 (7)
C20	0.0444 (12)	0.0253 (9)	0.0323 (9)	−0.0019 (8)	−0.0002 (9)	−0.0058 (8)
N21	0.0429 (10)	0.0319 (8)	0.0284 (8)	0.0021 (7)	0.0025 (7)	−0.0039 (7)
C22	0.0369 (11)	0.0293 (9)	0.0288 (9)	−0.0010 (8)	0.0053 (8)	0.0017 (7)
C23	0.0303 (9)	0.0228 (8)	0.0284 (9)	−0.0008 (7)	0.0011 (7)	−0.0022 (7)
O24	0.0350 (7)	0.0406 (8)	0.0216 (6)	0.0003 (6)	−0.0003 (5)	0.0052 (5)
C25	0.0353 (11)	0.0436 (11)	0.0279 (9)	0.0009 (9)	0.0001 (8)	−0.0011 (8)

### Geometric parameters (Å, °)

**Table:**

C1—N10	1.324 (2)	C13—C14	1.381 (3)
C1—C2	1.440 (2)	C13—H13	0.9500
C1—C11	1.482 (2)	C14—F17	1.356 (2)
C2—N3	1.325 (2)	C14—C15	1.378 (3)
C2—C18	1.486 (2)	C15—C16	1.387 (2)
N3—C4	1.362 (2)	C15—H15	0.9500
C4—C5	1.414 (2)	C16—H16	0.9500
C4—C9	1.416 (2)	C18—C19	1.388 (2)
C5—C6	1.360 (3)	C18—C23	1.391 (2)
C5—H5	0.9500	C19—C20	1.382 (3)
C6—C7	1.423 (3)	C19—H19	0.9500
C6—H6	0.9500	C20—N21	1.338 (3)
C7—O24	1.363 (2)	C20—H20	0.9500
C7—C8	1.369 (2)	N21—C22	1.338 (2)
C8—C9	1.410 (2)	C22—C23	1.384 (3)
C8—H8	0.9500	C22—H22	0.9500
C9—N10	1.363 (2)	C23—H23	0.9500
C11—C16	1.398 (2)	O24—C25	1.426 (2)
C11—C12	1.400 (2)	C25—H25A	0.9800
C12—C13	1.382 (2)	C25—H25B	0.9800
C12—H12	0.9500	C25—H25C	0.9800
			
N10—C1—C2	120.82 (15)	C12—C13—H13	120.7
N10—C1—C11	115.80 (15)	F17—C14—C15	118.43 (16)
C2—C1—C11	123.35 (15)	F17—C14—C13	118.67 (16)
N3—C2—C1	121.23 (15)	C15—C14—C13	122.89 (16)
N3—C2—C18	115.13 (15)	C14—C15—C16	117.77 (17)
C1—C2—C18	123.52 (15)	C14—C15—H15	121.1
C2—N3—C4	117.89 (15)	C16—C15—H15	121.1
N3—C4—C5	120.10 (16)	C15—C16—C11	121.39 (16)
N3—C4—C9	120.69 (15)	C15—C16—H16	119.3
C5—C4—C9	119.16 (16)	C11—C16—H16	119.3
C6—C5—C4	120.00 (17)	C19—C18—C23	117.26 (16)
C6—C5—H5	120.0	C19—C18—C2	121.15 (16)
C4—C5—H5	120.0	C23—C18—C2	121.47 (15)
C5—C6—C7	120.69 (16)	C20—C19—C18	118.88 (17)
C5—C6—H6	119.7	C20—C19—H19	120.6
C7—C6—H6	119.7	C18—C19—H19	120.6
O24—C7—C8	125.10 (17)	N21—C20—C19	124.49 (17)
O24—C7—C6	114.32 (15)	N21—C20—H20	117.8
C8—C7—C6	120.58 (16)	C19—C20—H20	117.8
C7—C8—C9	119.36 (16)	C20—N21—C22	116.18 (16)
C7—C8—H8	120.3	N21—C22—C23	123.54 (17)
C9—C8—H8	120.3	N21—C22—H22	118.2
N10—C9—C8	119.04 (15)	C23—C22—H22	118.2
N10—C9—C4	120.78 (15)	C22—C23—C18	119.64 (16)
C8—C9—C4	120.17 (15)	C22—C23—H23	120.2
C1—N10—C9	118.06 (15)	C18—C23—H23	120.2
C16—C11—C12	118.64 (16)	C7—O24—C25	116.56 (14)
C16—C11—C1	122.23 (15)	O24—C25—H25A	109.5
C12—C11—C1	119.03 (15)	O24—C25—H25B	109.5
C13—C12—C11	120.66 (16)	H25A—C25—H25B	109.5
C13—C12—H12	119.7	O24—C25—H25C	109.5
C11—C12—H12	119.7	H25A—C25—H25C	109.5
C14—C13—C12	118.61 (16)	H25B—C25—H25C	109.5
C14—C13—H13	120.7		
			
N10—C1—C2—N3	7.9 (3)	N10—C1—C11—C12	32.8 (2)
C11—C1—C2—N3	−170.22 (16)	C2—C1—C11—C12	−149.01 (16)
N10—C1—C2—C18	−167.94 (16)	C16—C11—C12—C13	−0.6 (3)
C11—C1—C2—C18	13.9 (3)	C1—C11—C12—C13	−177.19 (16)
C1—C2—N3—C4	−5.4 (2)	C11—C12—C13—C14	−0.8 (3)
C18—C2—N3—C4	170.78 (15)	C12—C13—C14—F17	−177.56 (16)
C2—N3—C4—C5	−178.67 (16)	C12—C13—C14—C15	2.1 (3)
C2—N3—C4—C9	−1.1 (2)	F17—C14—C15—C16	177.79 (16)
N3—C4—C5—C6	176.67 (16)	C13—C14—C15—C16	−1.9 (3)
C9—C4—C5—C6	−1.0 (3)	C14—C15—C16—C11	0.4 (3)
C4—C5—C6—C7	−0.7 (3)	C12—C11—C16—C15	0.8 (3)
C5—C6—C7—O24	−178.82 (16)	C1—C11—C16—C15	177.30 (16)
C5—C6—C7—C8	1.2 (3)	N3—C2—C18—C19	46.7 (2)
O24—C7—C8—C9	−179.94 (15)	C1—C2—C18—C19	−137.24 (18)
C6—C7—C8—C9	0.0 (3)	N3—C2—C18—C23	−129.12 (18)
C7—C8—C9—N10	177.21 (16)	C1—C2—C18—C23	47.0 (3)
C7—C8—C9—C4	−1.7 (3)	C23—C18—C19—C20	−1.1 (3)
N3—C4—C9—N10	5.7 (3)	C2—C18—C19—C20	−177.09 (17)
C5—C4—C9—N10	−176.72 (16)	C18—C19—C20—N21	1.7 (3)
N3—C4—C9—C8	−175.42 (15)	C19—C20—N21—C22	−1.0 (3)
C5—C4—C9—C8	2.2 (3)	C20—N21—C22—C23	−0.1 (3)
C2—C1—N10—C9	−3.2 (2)	N21—C22—C23—C18	0.6 (3)
C11—C1—N10—C9	175.05 (14)	C19—C18—C23—C22	0.1 (3)
C8—C9—N10—C1	177.83 (15)	C2—C18—C23—C22	176.04 (17)
C4—C9—N10—C1	−3.2 (2)	C8—C7—O24—C25	−3.7 (3)
N10—C1—C11—C16	−143.69 (17)	C6—C7—O24—C25	176.37 (16)
C2—C1—C11—C16	34.5 (2)		

### Hydrogen-bond geometry (Å, °)

**Table:**

*D*—H···*A*	*D*—H	H···*A*	*D*···*A*	*D*—H···*A*
C15—H15···N21^i^	0.95	2.44	3.368 (3)	165

Symmetry codes: (i) −*x*, −*y*, −*z*+1.
